# The morphogen Decapentaplegic employs a two-tier mechanism to activate target retinal determining genes during ectopic eye formation in Drosophila

**DOI:** 10.1038/srep27270

**Published:** 2016-06-07

**Authors:** Poonam Aggarwal, Jayati Gera, Lolitika Mandal, Sudip Mandal

**Affiliations:** 1 Molecular Cell and Developmental Biology Laboratory, Department of Biological Sciences, Indian Institute of Science Education and Research Mohali, Knowledge City, Sector 81, Mohali, Punjab 140306, India; 2 Developmental Genetics Laboratory, Department of Biological Sciences, Indian Institute of Science Education and Research Mohali, Knowledge City, Sector 81, Mohali, Punjab 140306, India

## Abstract

Understanding the role of morphogen in activating its target genes, otherwise epigenetically repressed, during change in cell fate specification is a very fascinating yet relatively unexplored domain. Our *in vivo* loss-of-function genetic analyses reveal that specifically during ectopic eye formation, the morphogen Decapentaplegic (Dpp), in conjunction with the canonical signaling responsible for transcriptional activation of retinal determining (RD) genes, triggers another signaling cascade. Involving dTak1 and JNK, this pathway down-regulates the expression of polycomb group of genes to do away with their repressive role on RD genes. Upon genetic inactivation of members of this newly identified pathway, the canonical Dpp signaling fails to trigger RD gene expression beyond a threshold, critical for ectopic photoreceptor differentiation. Moreover, the drop in ectopic RD gene expression and subsequent reduction in ectopic photoreceptor differentiation resulting from inactivation of dTak1 can be rescued by down-regulating the expression of polycomb group of genes. Our results unravel an otherwise unknown role of morphogen in coordinating simultaneous transcriptional activation and de-repression of target genes implicating its importance in cellular plasticity.

Cell fate specification and patterning within a tissue, are achieved by positional information provided by the concentration gradients of a group of signaling molecules termed morphogens^1^. These molecules impart an instructive role by transcriptional regulation of their target genes in the receiving cells depending on the relative position of these cells within the morphogenetic field^2^. Experimental evidences from both vertebrate and invertebrate model organisms have established that although the signaling pathway triggered by a morphogen is highly conserved^3^,^4^, the same extracellular signal elicits unique cell specific responses. This pleiotropic response evoked in different cell types is determined by the epigenetic modifications present within the genome^5^,^6^ and by the gamut of cell specific transcription factors present^7^,^8^. As a consequence, some genes that are responsive to a particular concentration of a morphogen signal in one cell might become refractory to the same signal in other cells.

Change in cell fate specification, however, poses a unique challenge to morphogen activity. It creates an exceptional situation wherein a morphogen might need to turn on its target genes that were hitherto kept silent in a cell. This demands that apart from transcriptional activation of target genes, epigenetic repression that renders the genes refractory to morphogen signaling needs to be erased. Understanding the mechanism by which both the things are coordinated represents an important aspect of morphogen activity that needs better insight.

One of the best worked out experimental models for understanding the biology of alteration in cell fate specification has been the larval imaginal discs of *Drosophila melanogaster.* Imaginal discs are sacs of epithelial cells present in larva that serve as anlagen of adult cuticular structures such as wings, legs, antenna and eye. Studies that involved transplanting imaginal disc fragments into the abdomen of adult female flies revealed that even though these regenerating disc fragments when allowed to metamorphose generally differentiate into their corresponding adult structures^9^, in some rare instances they can undergo cell fate alteration by a process known as transdetermination to give rise to adult structures generally derived from other imaginal discs^10^,^11^. Subsequent studies revealed that similar change in cell fate specification could also be achieved by genetic means. Ectopic expression of many different selector genes, and homeotic genes in developing imaginal discs can directly change the fate of imaginal disc cells and thereby lead to the generation of disc-inappropriate structures^12^. For instance, while mis-expression of the HOX gene *antennapedia* in the antenna causes antenna to leg transformation^13^, ectopic expression of *vestigial* induces ectopic wing like structures in the eyes, legs and antenna^14^. Likewise, ectopic eyes are generated on different parts of adult flies by ectopic expression of genes that include *eyeless, eyes absent* and *twin of eyeless* in the developing imaginal discs^15^,^16^,^17^.

Intriguingly, the morphogens are known to play an important role during cell fate alteration in imaginal discs. It has been observed that ectopic expression of the morphogen Wingless (Wg) in leg imaginal discs can lead to leg to wing conversion^18^. Moreover, cells undergoing leg to wing conversion in regenerating leg disc fragments also express high levels of Wg^19^. In a similar manner, wing to eye conversion by ectopic *eyeless* expression has been found to be restricted to the cells of developing wing disc that express higher levels of the morphogens Decapentaplegic (Dpp) and Hedgehog (Hh)^20^. Based on previous results it is generally perceived that during this process of transdetermination the morphogens play a critical role in activating a set of target genes that are essential for the altered cell fate. However, it is yet not known whether while doing so they impart any role in releasing the epigenetic repression that normally maintained these target genes in a refractory state to the signal prior to cell fate alteration.

To address this issue, we employed the well-established method of induction of eyes in larval wing imaginal discs of *Drosophila* by ectopic *eyeless* expression^15^,^16^ as our experimental model. Previous studies have established that a complex gene regulatory network generally termed as the Retinal Determining (RD) network^21^,^22^ governs the early events associated with eye development in *Drosophila*. While removal of individual members of the RD network inhibits eye formation^23^,^24^,^25^, induced expression of these genes is sufficient to generate ectopic eyes in non-retinal tissues^15^,^16^,^17^,^26^. Genes of the RD network that primarily involve *eyeless (ey*)*, eyes absent (eya*)*, sine oculis (so*) and *dachshund (dac*) transcriptionally regulate each others expression by feed back loops^26^,^27^,^28^,^29^,^30^,^31^,^32^ and even the proteins physically interact with one another to trigger downstream target genes^27^,^32^,^33^. Importantly, it has also been demonstrated that activation of *dac* along with *so* and *eya* are also dependent on Dpp signaling^28^,^31^,^34^. Since activation of the RD gene regulatory network is equally essential for ectopic eye induction^29^, it is intuitively obvious that during induction of ectopic eyes in developing wing discs Dpp needs to turn on *dac, so* and *eya* in wing disc cells where they are not normally expressed. This in turn serves as a wonderful platform to investigate the role played by Dpp, if any, in modulating the epigenetic landscape to facilitate ectopic expression of these RD genes during ectopic eye induction.

The outcome of this study unravels the mechanism by which Dpp modulates the expression of its target RD genes during ectopic eye induction in wing discs of *Drosophila*. We show that besides transcriptional activation of RD genes by the stereotype canonical signaling, Dpp involves another pathway to expunge the existing epigenetic repression on these genes. This two-tiered mechanism employed by Dpp in regulating the ectopic expression of RD genes reveals an otherwise unknown phenomenon by which a morphogen can elicit both instructive and permissive roles to regulate the expression of its target genes.

## Results

**Figure 1:**
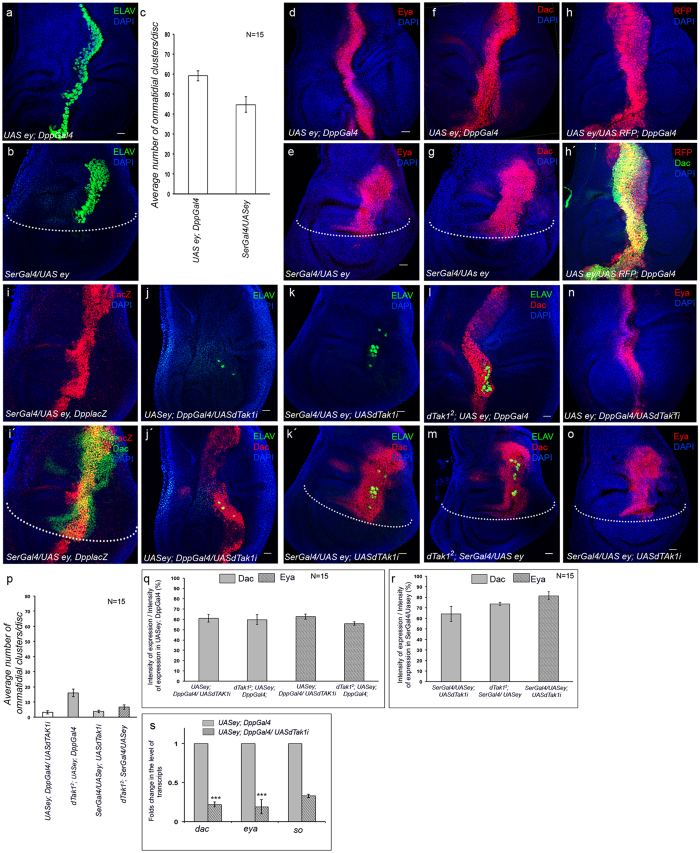
dTak1 regulates ectopic expression of RD genes in wing discs. Genotypes are as mentioned. For all wing discs anterior is to the left. Dotted line marks the Dorsal/Ventral boundary of the wing disc. (**a,b**) Ectopic expression of ELAV. (**c**) Quantification of the number of ectopic photoreceptor clusters. (**d–g**) Ectopic expression of Eya (**d,e**) and Dac (**f,g**). (**h–i´**) Overlap in the domains of Dac and Dpp expression. (**j–m**) Changes in the level of ectopic Dac expression (**j´,k´,l,m**) and number of ectopic photoreceptors. (**n,o**) Change in ectopic Eya expression. (**p**) Quantification of the number of ectopic photoreceptor clusters. (**q,r**) Quantification of the changes in fluorescence intensities of ectopic Dac and Eya expression. (**s**) Changes in levels of transcripts of RD genes. Scale = 20 μ.

For our studies we ectopically expressed the gene *eyeless (ey*), the most potent inducer of ectopic eye formation^16^, in developing wing discs employing the Gal4-UAS system^35^. We used two independent Gal4 drivers, *Dpp-Gal4* and *Ser-Gal4* to drive the expression of *UAS-ey*. In case of *Dpp-Gal4; UAS-ey* late third instar larval wing discs, differentiation of ectopic photoreceptors, as evidenced by immunostaining with antibodies against ELAV, was detected all along the A/P boundary (Fig. 1a,c), the domain of endogenous Dpp expression^36^. In contrast, ectopic photoreceptor differentiation in wing discs of late third instar *Ser-Gal4, UAS-ey* larvae was not observed in the entire dorsal compartment, where Ser is known to express^37^. Instead, we detected ELAV positive photoreceptors restricted to a topological area in the dorsal compartment that appeared to be along the A/P boundary (Fig. 1b,c). Immunostaining of these wing discs with antibodies against the two RD proteins Dac and Eya further revealed that the observed domain of ectopic photoreceptor differentiation was defined by ectopic expression domains of these two RD genes *eya* and *dac* (Fig. 1d–g). To better map the domain of ectopic Dac and Eya expression, we analyzed the domain of their expression with respect to that of Dpp along the A/P boundary. In both the cases we observed that the domain of Dac expression actually overlapped with the domain of elevated Dpp expression along A/P boundary (Fig. 1h–i´) generally observed in these discs upon ectopic expression of *ey*. RT-PCR analysis revealed that the other RD gene, *so*, also got ectopically expressed in these discs (Fig. S1a). All these results corroborate previous observations that established a synergistic role of canonical Dpp signaling with Eyeless in transcriptional activation of these three RD genes^28^,^31^,^34^ that constitute a complex RD gene regulatory network controlling the specification of eye primordia.

We argued that formation of ectopic eyes, however, is a more complicated process than normal eye differentiation. While during normal eye differentiation Dpp along with Ey has to activate the RD genes in cells otherwise determined to form photoreceptors, during ectopic eye induction they need to be turned on in cells that are not normally destined to form photoreceptors. We, therefore, ventured to explore whether Dpp plays any role beyond its expected function to ectopically activate RD genes in non-neuronal precursor cells during ectopic eye formation.

### dTak1 regulates the expression of RD genes critical for ectopic eye formation

Studies involving mammalian cell lines and *in vivo* model organisms have demonstrated that members of the TGF-β family of proteins, including Dpp, evoke various cellular responses by activation of the TGF-β activated Kinase 1 (Tak1)^38^,^39^,^40^. To determine the involvement of the *Drosophila Tak1 (dTak1*) in ectopic eye formation, we knocked down the expression of *dTak1* (Fig. S1b) in wing discs undergoing ectopic eye differentiation. This resulted in a significant reduction in the number of ectopic photoreceptors (Fig. 1j,k,p). Analogous results were observed when ectopic eyes were generated in wing discs of *dTak1* mutants (Fig. 1l,m,p). The failure in photoreceptor differentiation, however, was not due to drastic drop in ectopic expression of the RD proteins Eya and Dac as observed upon attenuating either *thickveins (tkv*) (Fig. S1c–e,j) or *Mother against dpp (Mad*) (Fig. S1f–j), the two members of the canonical Dpp signaling pathway. Rather, we observed reduced levels of ectopic expression of these two RD proteins, Dac and Eya, in wing discs of both *Dpp-Gal4; UAS-ey* and *Ser-Gal4, UAS-ey* late third instar larvae upon attenuating the activity of dTak1 (Fig. 1j´) without any alteration in the domain of their ectopic expression (Fig. S1k,l). Quantitative analyses of fluorescence intensity within the Dac and Eya expression domains revealed that compared to their respective controls there was around 30–40 percent drop in the levels of Dac and Eya expression upon attenuating dTak1 function (Fig. 1q,r). In consistence with these observations, results of qRT-PCR analyses also exhibited a drastic decrease in the level of ectopic transcripts of the three RD genes (Figs 1s and S1m). Together, these results suggested a threshold level of ectopic expression of RD genes to be critical for ectopic eye formation; and since that level was not achieved upon attenuation of dTak1 activity, ectopic photoreceptors failed to differentiate.

### dTak1 functions downstream of Dpp independent of the canonical pathway

**Figure 2:**
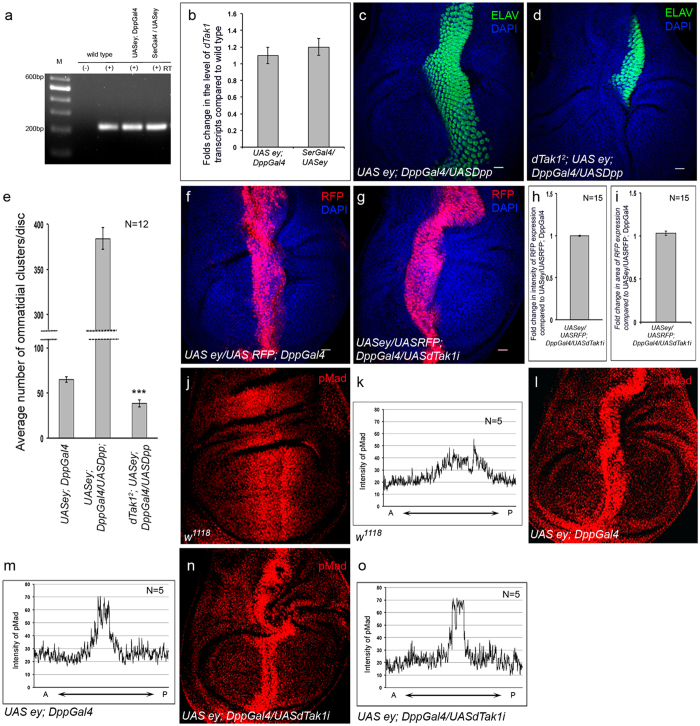
dTak1 functions downstream of Dpp during ectopic eye induction. Genotypes are as mentioned. For all wing discs anterior is to the left. (**a**) Expression of *dTak1* transcripts. (**b**) Changes in the levels of *dTak1* transcripts. (**c,d**) Ectopic expression of ELAV. (**e**) Quantification of the number of ectopic photoreceptor clusters. (**f,g**) Reporter RFP expression for Dpp. (**h,i**) Quantification of the changes in fluorescence intensity (**h**) and area (**i**) of reporter RFP expression. (**j–o**) Expression of pMad in wing discs (**j,l,n**) and their corresponding average intensity profile (**k,m,o**). Scale = 20 μ.

To further investigate the involvement of dTak1, we analyzed its expression in wild-type wing discs vis-à-vis in wing discs expressing ectopic *eyeless*. Results of both RT-PCR and qRT-PCR analyses did not reveal any appreciable change in the levels of *dTak1* expression (Fig. 2a,b). To determine whether dTak1 functions in a Dpp dependent manner we performed genetic epistasis experiments. As evident from Fig. 2c,e, overexpression of Dpp in wing discs undergoing ectopic eye differentiation lead to a robust increase in the number of ectopic photoreceptors. However, this increase got drastically suppressed in *dTak1* mutant background (Fig. 2d,e). In contrast, the levels of Dpp expression, as revealed by reporter RFP expression, remained unaltered upon attenuating *dTak1* function (Fig. 2f,g). Quantitative analyses did not exhibit any significant changes either in the intensity (Fig. 2h) or in the domain (Fig. 2i) of RFP expression. Together, these results established that dTak1 acts downstream of Dpp.

Next, we monitored the expression of phosphorylated Mad (pMad) to investigate whether knocking down *dTak1*, by any chance had affected canonical Dpp signaling. As evident from Fig. 2l,m, compared to wild type wing discs (Fig. 2j,k) there was an increase in levels of pMad expression in wing discs of *Dpp-Gal4; UAS ey* larvae. Interestingly, the increased level of pMad expression remained unaltered in discs where *dTak1* expression was compromised (Fig. 2n,o). Furthermore, expression of Dac and Eya (Fig. S2a–e) and subsequent differentiation of ommatidial clusters during normal eye development (Fig. S2f–h) remained unaffected in eye discs of *dTak1* mutants suggesting that dTak1 had no role during this process. Collectively, these results established that specifically during ectopic eye induction, besides transcriptional activation of RD genes by canonical signaling, Dpp also employed another signaling pathway mediated by *dTak1* to control ectopic expression of RD genes.

### dTak1 activates Jun-N-terminal Kinase (JNK) to modulate expression of RD genes

**Figure 3:**
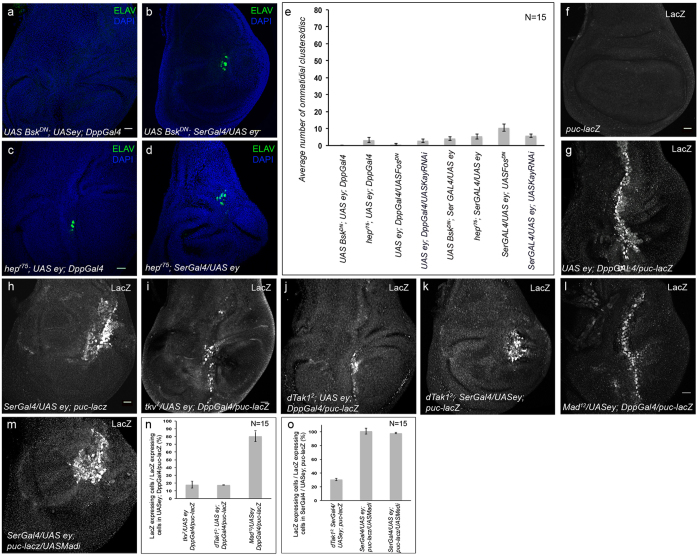
dTak1 activates JNK pathway during ectopic eye induction. Genotypes are as mentioned. For all wing discs anterior is to the left. (**a–d**) Ectopic expression of ELAV. (**e**) Quantification of the number of ectopic photoreceptor clusters. (**f**) Expression of reporter puc-lacZ in wild-type wing disc. (**g,h**) Ectopic expression of reporter puc-lacZ. (**i–m**) Changes in the level of ectopic puc-lacZ expression. (**n,o**) Quantification of the changes in ectopic puc-lacZ expression. Scale = 20 μ.

To identify the downstream target through which dTak1 regulates the expression of RD genes, we systematically eliminated components of different signal transduction pathways known to be activated by dTak1 (Table S1)^41^, and screened for their role in ectopic eye induction. A dominant negative form of *basket (bsk;* JNK in flies), when co-expressed with *eyeless*, dramatically reduced the number of ectopic photoreceptors in late third instar larval wing discs (Fig. 3a,b,e). Comparable decrease in ectopic eye induction was also observed in wing discs mutant for *hemipterous (hep*; JNKK in flies) (Fig. 3c–e). Likewise, knocking down Kayak (Kay; Fos in flies) (Fig. S3a), one of the downstream transcriptional regulators of JNK pathway, resulted in a block in ectopic photoreceptor differentiation (Figs S3b,c and 3e). Identical drop in differentiation of ectopic photoreceptors was obtained upon co-expressing a dominant negative form of *fos* with *eyeless* (Figs S3d,e and 3e). However, knocking down the other transcriptional activator Foxo or generating ectopic eyes in wing discs heterozygous mutant for *foxo*, had no significant effect on ectopic photoreceptor differentiation (Fig. S3f–j) thereby indicating that ectopic photoreceptor differentiation regulated by JNK to be mediated by AP1, a heterodimer formed by dJun and dFos.

Furthermore, we monitored JNK activity by the expression of the gene *puckered (puc*), a direct downstream target of JNK signaling. In contrast to wild type late third instar larval wing discs where no puc-lacZ expression was observed in the pouch region (Fig. 3f) induction of ectopic eyes caused ectopic puc-lacZ expression specifically in the areas where ectopic eye differentiation were observed (Fig. 3g,h). However, this puc-lacZ expression got significantly reduced when ectopic eyes were generated in wing discs of larvae heterozygous mutant for *tkv* loss of function allele*, tkv*^7^ (Fig. 3i,n). Similar results were observed when ectopic eyes were generated in *dTak1* mutant wing discs (Fig. 3j,k,n,o) as well as upon co-expressing a dominant negative form of *bsk* (Fig. S3k,l). However, the puc-lacZ expression remained unaltered when ectopic eyes were induced in wing discs mutant for Mad or upon knocking down Mad (Fig. 3l–o). Similarly knocking down Medea (Med), the partner of Mad for transcriptional activation of Dpp target genes, did not alter the puc-lacZ expression (Fig. S3m) and in the process ruled out the chance of any involvement of canonical Dpp signaling in activation of JNK.

**Figure 4:**
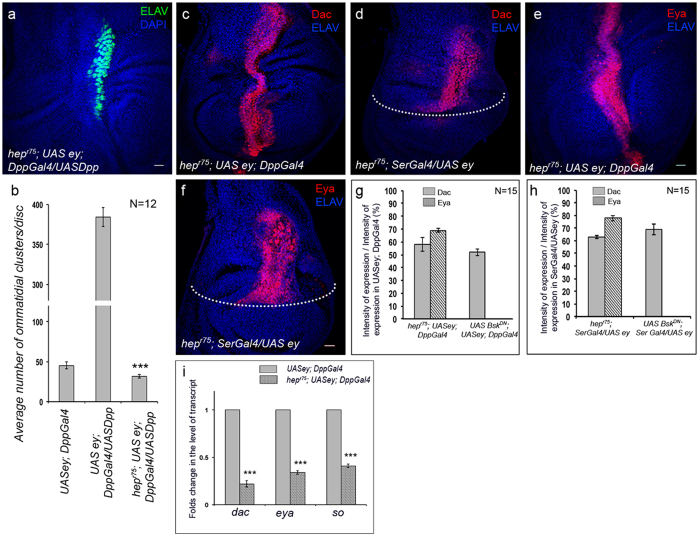
dTak1 modulates the ectopic expression of RD genes through JNK pathway. Genotypes are as mentioned. For all wing discs anterior is to the left. Dotted line marks the Dorsal/Ventral boundary of the wing disc. (**a**) Ectopic expression of ELAV. (**b**) Quantification of the number of ectopic photoreceptor clusters. (**c,d**) Changes in the level of ectopic Dac expression. (**e,f**) Changes in the level of ectopic Eya expression. (**g,h**) Quantification of the changes in fluorescence intensities of ectopic Dac and Eya expression. (**i**) Changes in levels of transcripts of RD genes. Scale = 20 μ.

To determine the hierarchical relationship between Dpp and JNK pathways we then performed genetic epistatis analyses. Our results revealed that the increase in number of ectopic photoreceptors as observed upon over-expressing Dpp (Fig. 2c) was significantly reduced in *hep* mutant background (Fig. 4a,b). However, the expression of Dpp as revealed by reporter RFP expression remained unaltered when ectopic eyes were induced in wing discs mutant for *hep* (Fig. S4a–d). Based on the results of mutant analyses, expression studies and genetic epistasis analysis we conclude that during ectopic eye formation Dpp triggers JNK signaling by activating dTak1, not by the canonical pathway involving Mad.

Consistent with these results we observed a drop in the levels of ectopic Dac and Eya expression when ectopic eyes were induced in wing discs of *hep* mutants (Fig. 4c–f) or upon over-expressing dominant negative form of *bsk* (Fig. S4e,f). Interestingly in both instances, the domains of Dac expression remained unaltered (Fig. S4g,h). Quantitative analyses of the fluorescence intensities of Dac and Eya expression (Fig. 4g,h) and that of the transcript levels of the three RD genes by qRT-PCR (Figs 4i and (S4i) revealed identical reduction as observed for knocking down *dTak1*. Together, all these results firmly put the JNK pathway as component of the non-canonical Dpp signaling, downstream of dTak1, involved in modulating the expression of RD genes during ectopic eye induction.

### JNK signaling modulates expression of RD genes by regulating PcG genes

Independent studies that involved ChiP-qPCR and *in silico* analysis of the *Drosophila* genome, have identified *dac, so* and *eya* as direct targets for Polycomb group (PcG) of proteins^42^,^43^,^44^ that regulate lineage choices by repressing gene expression by modifying chromatin architecture^45^. Since the RD genes are not normally expressed in the wing discs (Fig. S5a,b), except for Dac in a rudimentary region in anterior compartment (arrow in Fig. S5a), we were curious to investigate the involvement of the non-canonical Dpp signaling in releasing any PcG mediated repression of these RD genes during ectopic eye induction.

**Figure 5:**
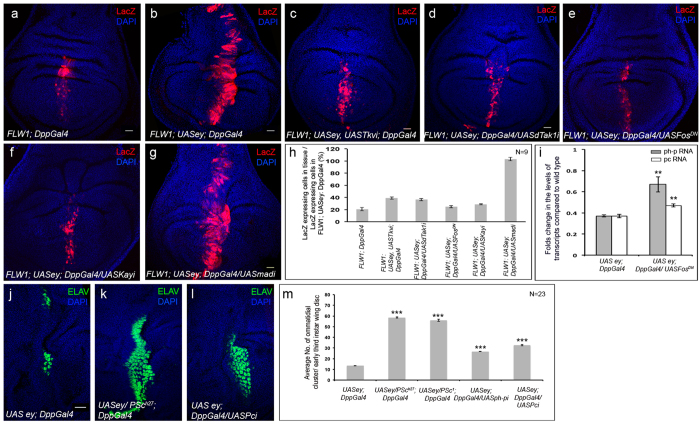
Involving JNK signaling pathway Dpp represses PcG genes. Genotypes are as mentioned. For all wing discs anterior is to the left. (**a–g**) Expression of polycomb reporter lacZ. (**h**) Quantification of the changes in polycomb reporter lacZ expression. (**i**) Changes in the levels of *ph-p* and *Pc* transcripts. (**j–l**) Ectopic photoreceptor differentiation in early third instar larval wing discs. (**m**) Quantification of the number of ectopic photoreceptor clusters. Scale = 20 μ.

To start with, we used a polycomb reporter line FLW1^46^ that expresses lacZ when polycomb proteins are down regulated. Compared to very low level of reporter lacZ expression in late third instar larval wing discs of *Dpp-Gal4* larvae (Fig. 5a), a significant increase in lacZ expression was observed in wing discs of late third instar *Dpp-Gal4; UAS-ey* larvae (Fig. 5b) suggesting down regulation of PcG activity. Importantly, this enhancement in lacZ expression got suppressed upon knocking down either *tkv* (Fig. 5c,h) or *dTak1* (Fig. 5d,h). Similar results were obtained when JNK signaling was blocked at the level of dFos (Fig. 5e,f,h). In contrast, the lacZ expression remained unaltered upon knocking down Mad (Fig. 5g,h and S5c,d). Finally, we found that the drop in the level of transcripts of PcG genes, *polyhomeotic proximal (ph-p*) and *polycomb (Pc*), as observed in late third instar wing discs of *Dpp-Gal4; UAS-ey* larvae got significantly restored upon attenuating the activity of dFos (Fig. 5i). In sum, these results suggested the involvement of non-canonical Dpp signaling in down regulating the expression of PcG genes during ectopic eye induction.

For functional correlation we analyzed the number of differentiating photoreceptors when ectopic eyes were induced in wing discs heterozygous mutant for the polycomb gene *Posterior sex comb (Psc*) or independently knocked down for the expression of *ph-p* (Fig. S5e) and *Pc* (Fig. S5f) genes. For these analyses we resorted to early third instar wing discs to avoid gross morphological distortions associated with late third instar wing discs of these genotypes undergoing ectopic eye differentiation. Compared to control (Fig. 5j), we detected a remarkable enhancement in the number of ectopic photoreceptors when expression of individual PcG genes was attenuated (Fig. 5k–m).

**Figure 6:**
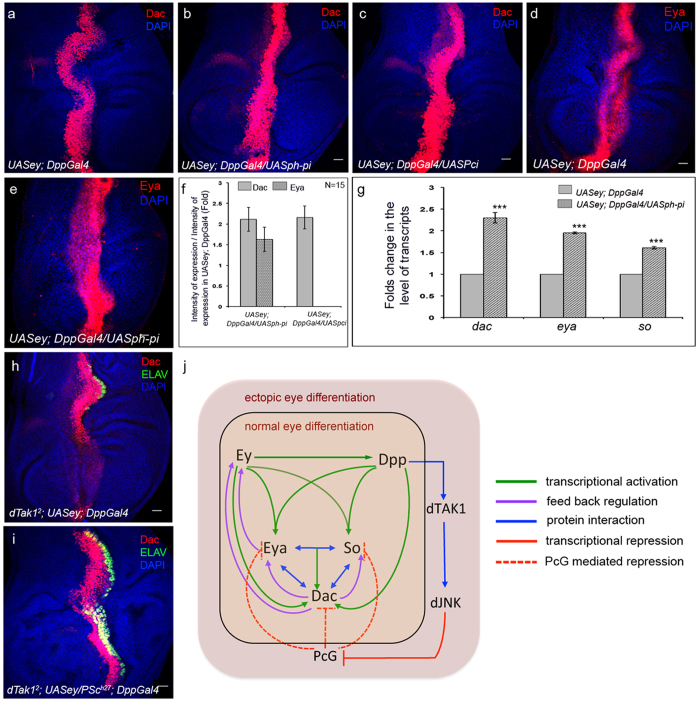
dTak1 mediated repression of PcG genes de-represses RD gene expression during ectopic eye induction. Genotypes are as mentioned. For all wing discs anterior is to the left. (**a,c**) Changes in the level of ectopic Dac expression (**b,c**) when compared to control (**a**). (**d,e**) Change in the level of ectopic Eya expression (**e**) when compared to control (**d**). (**f**) Quantification of the changes in fluorescence intensities of ectopic Dac and Eya expression. (**g**) Changes in the levels of transcripts of RD genes. (**h,i**) Expression of ectopic Dac and ELAV. (**j**) Genetic pathway elucidating the role of Dpp signaling in regulating ectopic expression of RD genes during ectopic eye induction. Scale = 20 μ.

Next we checked for alteration in the levels of ectopic Dac and Eya expression, if any, in these wing discs knocked down for PcG genes. Knocking down *ph-p* and *Pc,* exhibited a detectable increase in levels of Dac (Fig. 6b,c,f) and Eya (Fig. 6e,f) expression when compared to their respective controls (Fig. 6a,d). Despite this increase in the level of expression, the domain of Dac and Eya expression remained unaltered (Fig. S6a). qRT-PCR analyses also revealed a significant up regulation in transcript levels of the three RD genes (Fig. 6g). Finally, to establish the involvement of PcG proteins in modulating the expression of RD genes by dTak1 mediated Dpp signaling, we wanted to determine if the decrease in Dac expression as observed in *dTak1* mutants (Fig. 6h) could be rescued by down regulating PcG activity. Indeed, generating ectopic eyes in wing discs double mutant for *dTak1* and *Psc* resulted in a dramatic increase in Dac expression (Fig. 6i) and a concomitant recovery in the number of ectopic photoreceptors (compare Fig. 6i with 6h). Together, these results established that during ectopic eye induction Dpp signaling mediated by dTak1 and JNK was instrumental in down regulating the expression of PcG genes and in the process released PcG mediated repression on RD genes.

## Discussion

The morphogen Dpp plays essential roles in regulating a wide range of developmental processes throughout fly development. As applicable for all other morphogens, the known responses to Dpp can be attributed to direct transcriptional activation or repression of its target genes. For instance, genetic and molecular evidences have linked canonical Dpp signaling with transcriptional activation of *eya, so* and *dac* during normal as well as ectopic eye development^27^,^28^,^31^. These three genes along with *ey* regulate each others’ expression by multiple feed back loops and the proteins even physically interact among themselves to constitute the complex RD gene regulatory network (Fig. 6j; modified from^28^,^33^) that commits a population of cells to adopt an eye tissue fate. In contrast, in wing discs Dpp indirectly controls target gene expression by down regulating the expression of *brinker*^47^, which codes for a transcriptional repressor of Dpp target genes^48^. In this study we provide *in vivo* genetic evidence of yet another mechanism by which Dpp regulates the expression of its target RD genes independent of its bona fide transcriptional regulator specifically during induction of ectopic eyes. We show that apart from previously known transcriptional activation of its target RD genes, during this process, Dpp simultaneously triggers another signaling cascade that involves dTak1-mediated activation of JNK. In turn, activated JNK down regulates the expression of PcG genes to alleviate PcG mediated repression of its target RD genes (Fig. 6j) in non-retinal tissues. The failure of canonical Dpp signaling in triggering the expression of RD genes beyond a threshold critical for ectopic eye differentiation upon attenuating this pathway highlights the significance of this cascade in regulating ectopic RD gene expression. In this context it is important to note that reducing ectopic Dac expression below the threshold by alternate means also inhibits ectopic photoreceptor differentiation in a comparable manner (Fig. S6b–f). Summing up, this *in vivo* genetic analysis unravels the employment of a two-tier regulatory mechanism by Dpp in modulating the expression of target RD genes during eye induction in non-retinal tissue.

Our results show that compared to *eya* and *so*, changes in the level of *dac* expression are more intense when the activities of members of this newly identified pathway are genetically manipulated. Even though *dac*, like *so* and *eya*, is a target of PcG mediated repression, *dac* transcription can also be regulated by So and Eya (Fig. 6j). Therefore, it is possible that the overall reduction is an outcome of both the processes. On the flip side, de-repression of *dac* can also partially contribute in regulating *eya* and *so* transcription by employing the feed back loop. Given the intricate nature of interaction of these three genes it is rather unfeasible to delineate the contribution of these processes. However, by establishing the genetic link that connects Dpp and de-repression of its target genes in the chromatin level, this study paves the way for future molecular analysis of the involvement of cis-regulatory elements of these genes in PcG mediated repression.

Previous studies have implicated that the two morphogens, Dpp and Hh are critical in defining the cellular and molecular environment that supports eye formation in imaginal discs of *Drosophila*^20^,^31^. It has been demonstrated that while ectopic eyes, when induced in imaginal discs, are generally positioned along the domain of high Dpp and Hh activity, co-expression of *ey* with either *dpp* or *hh* can extend the domain of ectopic eye induction in developing wing discs. Contradicting this notion, a recent study demonstrated that not all cells within the Dpp expression domain are capable of supporting ectopic eye formation. Rather they identified discrete population of cells within the Dpp expression domain, generally termed as hot spots^15^, that have the cellular plasticity to form ectopic eyes. Our results, however, differ from this concept as we demonstrate that driving *UAS-ey* by *Dpp-Gal4* induces ectopic photoreceptor differentiation all along the Dpp expression domain (A/P boundary). Even in case of *Ser-Gal4, UAS-ey* wing discs we observed ectopic photoreceptor differentiation along the A/P boundary specifically in the Dpp expression domain within the dorsal compartment that spans beyond the identified hot spot region. However, the relevance of hot spots become apparent when we analyze the spatial distribution of ectopic photoreceptor differentiation in wing discs upon attenuating the newly identified signaling cascade. The limited number of ectopic photoreceptor differentiation associated with some of these cases indeed trace back to the identified hot spot regions, suggesting that these cells have greater developmental flexibility than other cells of the Dpp expression domain. In this context, it is important to note that as shown in Fig. 5a, the lacZ expression of PcG activator reporter FLW1 in *Dpp-Gal4* wing discs also correlate with one of the hot spots identified in the wing disc. Intriguingly, this correlation is not limited to wing discs but can also be seen in leg and haltere discs (Fig. S6g,h). Since lacZ expression of this polycomb reporter line actually reports low PcG activity^46^,^49^, it is quite evident that the developmental plasticity demonstrated by the cells of the hot spot region to adopt retinal fate, superseding their primary developmental instruction, is due to reduced level of PcG activity.

For our analyses we drove the expression of *UAS-ey* either by *Dpp-Gal4* or by *Ser-Gal4* from the first instar of larval development. In *Dpp-Gal4; UAS-ey* wing discs, as expected, we observed ectopic photoreceptor differentiation all along A/P boundary, the domain in which *Dpp-Gal4* is known to express^50^. However, in *Ser-Gal4, UAS-ey* wing discs we detected ectopic photoreceptor differentiation to be restricted to a topological area within the dorsal compartment that overlaps with the A/P boundary; not in the entire dorsal compartment where *Ser-Gal4* normally expresses^37^. Likewise, even though in both the cases ectopic *ey* expression lead to an up regulation in the level of Dpp expression as reported earlier (Fig. 6j)^20^ , the observed increase was specifically limited to the endogenous domain of Dpp expression along the A/P boundary. While the up regulation of Dpp in its endogenous domain does make sense for *Dpp-Gal4, UAS-ey* wing discs as ectopic *eyeless* expression was being driven particularly in the domain of normal Dpp expression, it is rather interesting to find that despite being induced to express in the entire dorsal compartment, *eyeless* was capable of enhancing Dpp expression only in its own endogenous domain. Since it has been demonstrated that wing to eye conversion by ectopic *eyeless* expression in developing wing disc require higher levels of both Dpp and Hh activity^20^, we believe that the reason for this spatial restriction might be due to the requirement of short range Hh signaling from the posterior compartment of wing disc that is known to regulate Dpp expression along the A/P border.

PcG proteins, long considered as epigenetic regulators that stably lock the expression state of Hox genes for an organism’s entire life span^51^ are also capable of tissue specific dynamic gene regulation during development^52^. The activation and dynamic regulation of genes repressed by PcG protein complexes can be achieved at different levels. In flies, while testis specific transcription factors are capable of counteracting PcG mediated gene silencing by selectively removing PcG protein complexes from promoters to trigger the expression of testis specific genes^53^, stress mediated activation of JNK in fragmented imaginal leg discs causes transcriptional down regulation of PcG genes to facilitate transdetermination^49^. Even during normal eye development in *Drosophila*, the developing R7 photoreceptors employ the PcG proteins to maintain just one of the alternative choices made during their commitment of fate^54^. Furthermore, PcG mediated epigenetic repression can also get erased by the activation of histone demethylases specific for H3K27. In particular, Nodal, a member of the TGF-β family, recruits Jmjd3 (a H3K27 demethylase) to its target loci by activated Smad2/3 to de-repress PcG activity in mouse embryonic stem cells^55^. Furthering the concept of dynamic regulation of PcG target genes, in this study we provide the genetic evidence of a newly identified mechanism that involves cross talk between TGF-β and JNK signaling pathways to counteract PcG mediated repression by down regulating the expression of PcG genes. Importantly, this role of Dpp in de-repressing RD genes is specifically associated with ectopic eye induction as it is not observed during normal photoreceptor differentiation or upon mere over-expression of Dpp (Fig. S6j–l). A logical next step, therefore, is to determine the mechanism that potentiates context dependent activation of this pathway by Dpp. Attenuating this pathway, however, does not lead to complete loss of expression of RD genes thereby indicating the presence of an yet to be identified mechanism involved in partial removal of PcG mediated repression to initiate ectopic expression of RD genes. In the light of the spatial restriction of the domain of ectopic eye formation in the A/P boundary it would be intriguing to determine whether Hh signaling plays any role in this process.

Efforts in developing strategies to alter the fate of adult cells have gained tremendous importance in the recent past, primarily because of their therapeutic application in regenerative medicine. Studies in this direction have evidenced that cell fate switching of adult cells is accomplished either by ectopic expression of specific transcription factor/s^56^,^57^ or even by modulating the expression levels of morphogens^58^. Moreover, switching of cell fate, has also been evidenced under certain pathophysiological conditions that include myofibroblastic transdifferentiation^59^ and Barrett’s disease^60^. Considering the conserved nature of morphogens and their signaling pathways between flies and vertebrates, determining whether morphogens employ similar kind of two-tier mechanism, as described here, in regulating their target genes during these processes would not only help us better understand cellular plasticity under diseased condition but would also contribute in designing better methods to induce cell fate switching for therapeutic purposes.

## Methods

### Fly stocks

For details of the fly stocks used, please refer to the Table S2 in supplemental materials and methods.

### Immunohistochemistry, confocal microscopy

All immunostainings were performed at least thrice (n = 15 per experiment) following the standard protocol. Details of primary and secondary antibodies used are provided in Table S3 of supplementary materials and methods. Confocal images were captured in LSM 780 (Carl Zeiss) and processed using Image J and Adobe Photoshop.

### Image analysis

Fluorescence intensity of expression was quantified in terms of gray value of Dac and Eya expressing area by using Image J. Each experiment was repeated thrice with appropriate controls and at least five images from each experiment were analyzed. Imaging was done with similar parameter settings. pMad activity was quantified in terms of pixel intensities using plot profile function of Image J.

### Area measurement

We used ImarisX64 and Image J for calculating the area of expressing cells and normalized with respect to total area of disc to account for variability in discs size. The calculated areas in experimental samples were compared to their corresponding controls to determine the ratio. The results shown here are outcome of three independent experiments. Ommatidial number was also calculated by using ImarisX64.

### Quantitative Analysis of transcripts

Transcript levels of *dtak1, tkv, dac, kay, eya, so, Pc* and *php* were analyzed by RT-PCR and qRT-PCR on RNA isolated from 50–60 wing discs. In all cases the expression levels were normalized with levels of *rp49* expression. Each experiment was repeated thrice with triplicates in each time. List of primers are in Table S4 of supplementary materials and methods.

### Statistical Analyses

Data are expressed as mean ± standard deviation (SD) of values from at least three independent experiments. Statistical analysis was performed using two-tailed Student’s t-test. P values of <0.01%; <0.001% and <0.0001%, mentioned as *, **, *** respectively were considered as statistically significant.

## Additional Information

**How to cite this article**: Aggarwal, P. *et al*. The morphogen Decapentaplegic employs a two-tier mechanism to activate target retinal determining genes during ectopic eye formation in Drosophila. *Sci. Rep.* **6**, 27270; doi: 10.1038/srep27270 (2016).

## Supplementary Material

Supplementary Information
