# Supplementary material for: The morphogen Decapentaplegic employs a two-tier mechanism to activate target retinal determining genes during ectopic eye formation in Drosophila
Source: Sci Rep. 2016 Jun 7;6:27270. doi: 10.1038/srep27270 (PMC4895176; doi:10.1038/srep27270)
Supplement: Supplementary Information [file srep27270-s1.pdf]

Supplementary Information

The morphogen Decapentaplegic employs a two-tier mechanism to activate target retinal determining genes during ectopic eye formation in Drosophila

Poonam Aggarwal[1], Jayati Gera[1], Lolitika Mandal[2] and Sudip Mandal[1]

[1] Molecular Cell and Developmental Biology Laboratory

[2] Developmental Genetics Laboratory

Department of Biological Sciences, Indian Institute of Science Education and Research Mohali, Knowledge City, Sector 81, Mohali, Punjab 140306, India

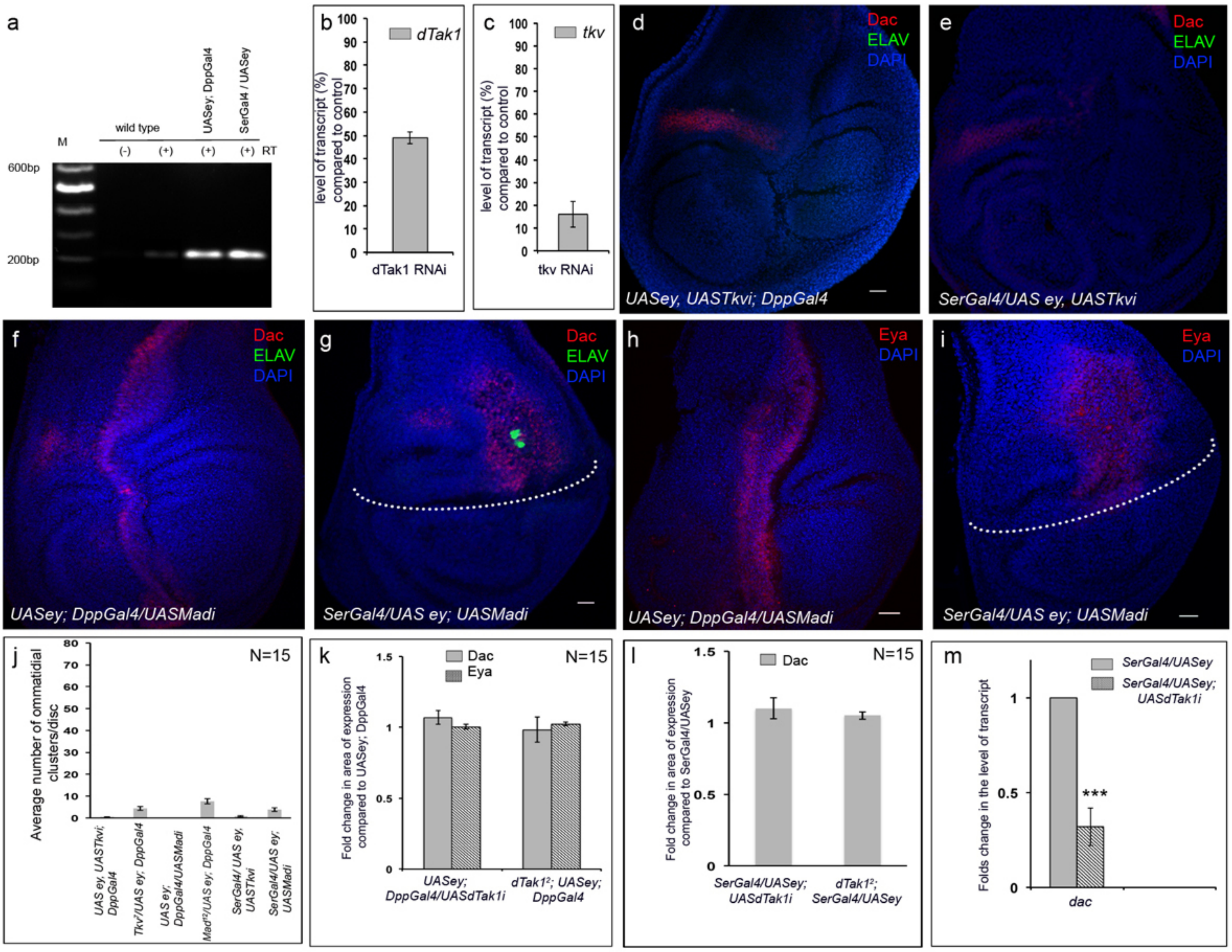

a
M
wild type
UASey; DppGal4
SerGal4 / UASey
(-)
(+)
(+)
(+)
RT
600bp
200bp
b
dTak1
level of transcript (%) compared to control
dTak1 RNAi
c
tkv
level of transcript (%) compared to control
tkv RNAi
d
Dac
ELAV
DAPI
UASey, UASTkvi; DppGal4
e
Dac
ELAV
DAPI
SerGal4/UAS ey, UASTkvi
f
Dac
ELAV
DAPI
UASey; DppGal4/UASMadi
g
Dac
ELAV
DAPI
SerGal4/UAS ey; UASMadi
h
Eya
DAPI
UASey; DppGal4/UASMadi
i
Eya
DAPI
SerGal4/UAS ey; UASMadi
j
N=15
Average number of ommatidial clusters/disc
UAS ey, UASTkvi; DppGal4
Tkv7/UAS ey; DppGal4
UAS ey; DppGal4/UASMadi
Mad12/UAS ey; DppGal4
SerGal4/ UAS ey, UASTkvi
SerGal4/UAS ey; UASMadi
k
Dac
Eya
N=15
Fold change in area of expression compared to UASey; DppGal4
UASey; DppGal4/UASdTak1i
dTak12; UASey; DppGal4
l
Dac
N=15
Fold change in area of expression compared to SerGal4/UASey
SerGal4/UASey; UASdTak1i
dTak12; SerGal4/UASey
m
SerGal4/UASey
SerGal4/UASey; UASdTak1i
Folds change in the level of transcript
***
dac

Figure S1

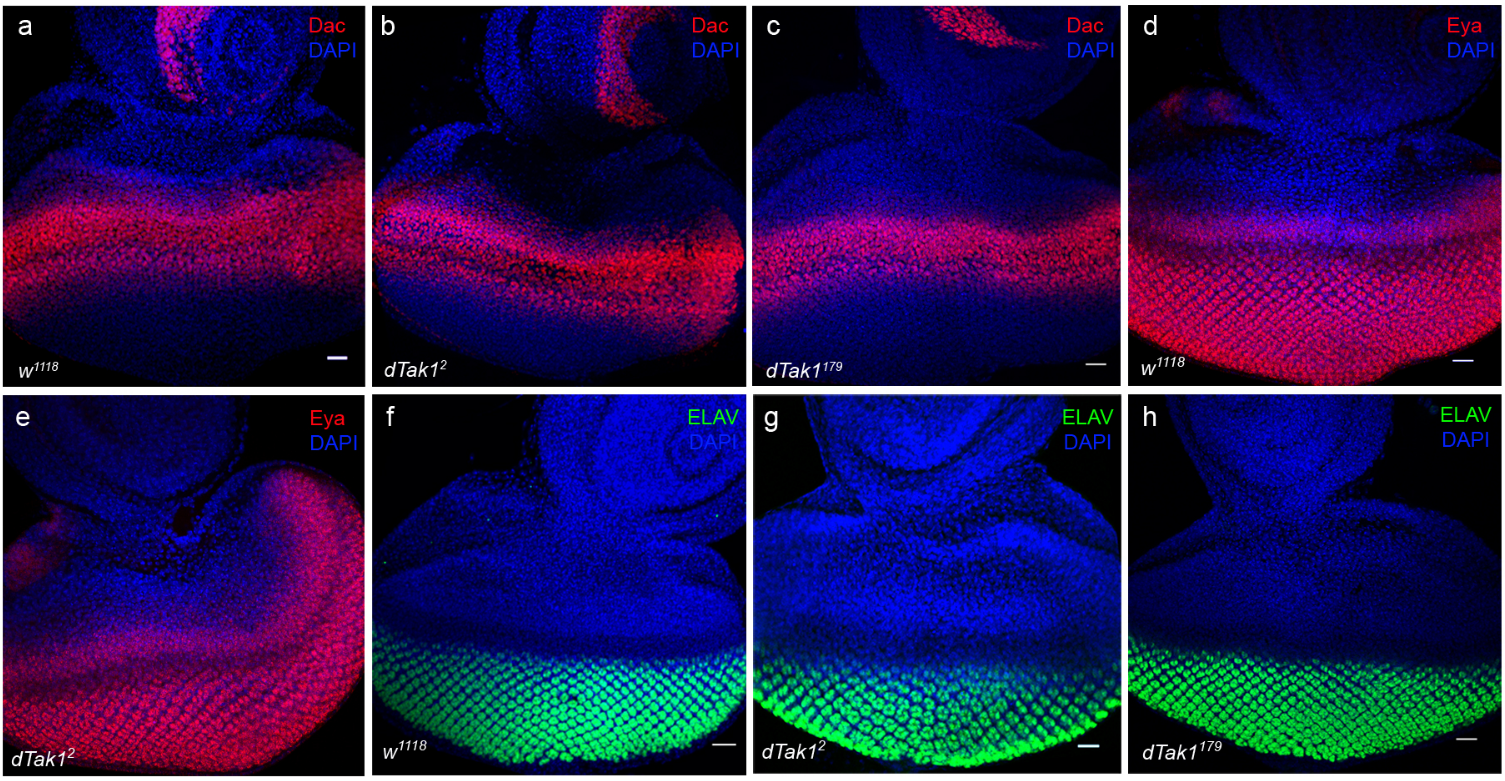

a
Dac
DAPI
w^{1118}
b
Dac
DAPI
$dTak1^{2}$
c
Dac
DAPI
$dTak1^{179}$
d
Eya
DAPI
w^{1118}
e
Eya
DAPI
$dTak1^{2}$
f
ELAV
DAPI
w^{1118}
g
ELAV
DAPI
$dTak1^{2}$
h
ELAV
DAPI
$dTak1^{179}$

Figure S2

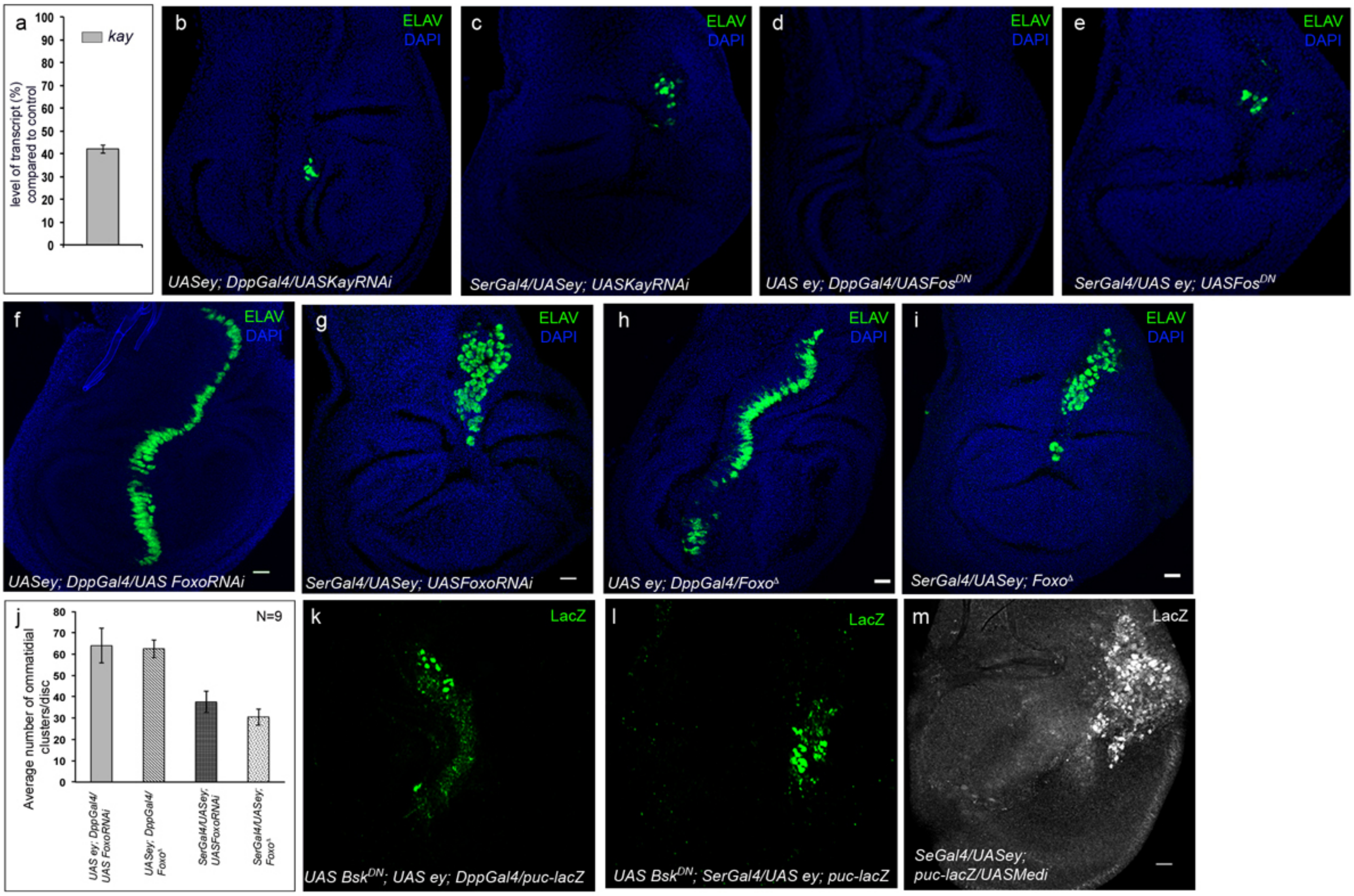
a
kay
level of transcript (%) compared to control
b
UASey; DppGal4/UASKayRNAi
c
SerGal4/UASey; UASKayRNAi
d
UAS ey; DppGal4/UASFosDN
e
SerGal4/UAS ey; UASFosDN
f
UASey; DppGal4/UAS FoxoRNAi
g
SerGal4/UASey; UASFoxoRNAi
h
UAS ey; DppGal4/FoxoΔ
i
SerGal4/UASey; FoxoΔ
ELAV
DAPI
j
N=9
Average number of ommatidial clusters/disc
UAS ey; DppGal4/ UAS FoxoRNAi
UASey; DppGal4/ FoxoΔ
SerGal4/UASey; UASFoxoRNAi
SerGal4/UASey; FoxoΔ
k
UAS BskDN; UAS ey; DppGal4/puc-lacZ
l
UAS BskDN; SerGal4/UAS ey; puc-lacZ
m
SeGal4/UASey; puc-lacZ/UASMedi
LacZ

Figure S3

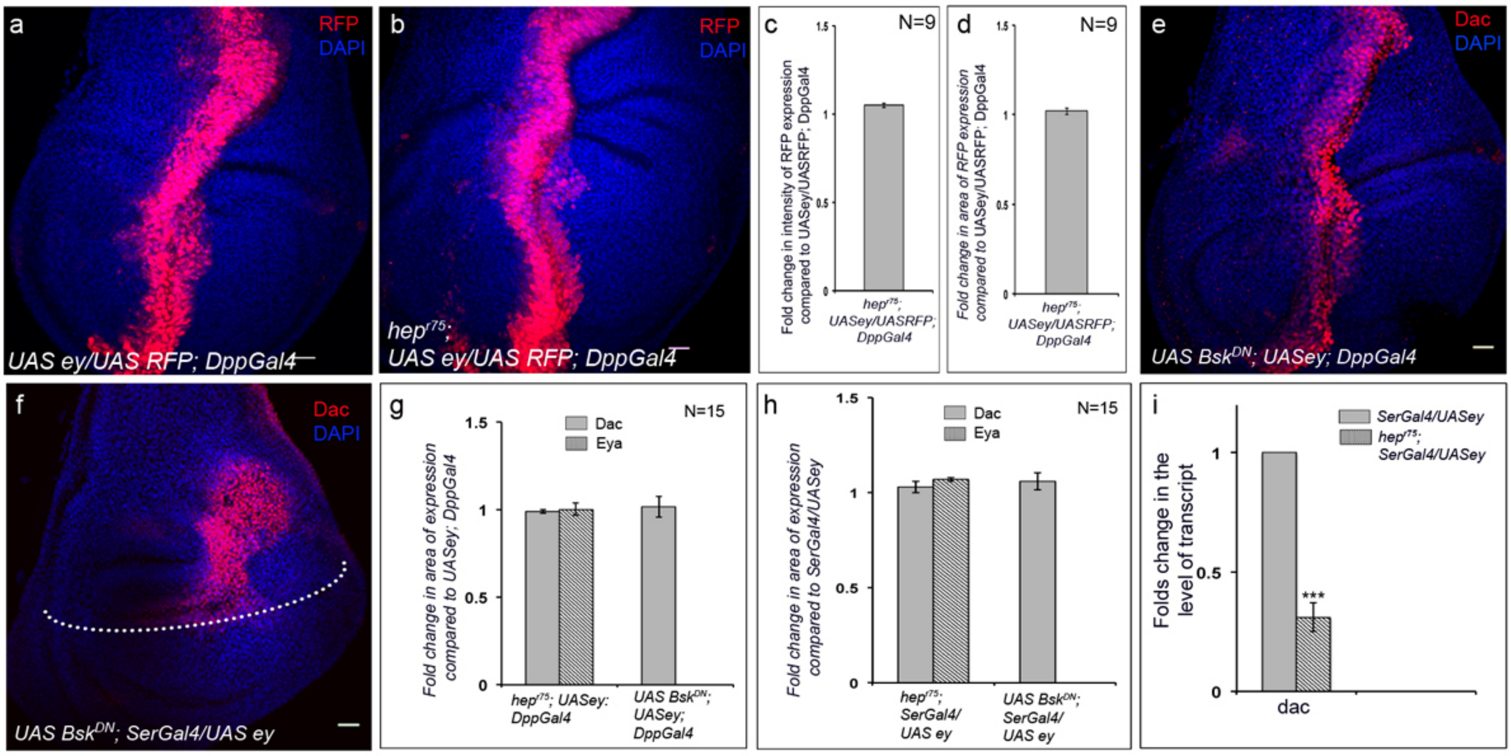
a
RFP
DAPI
UAS ey/UAS RFP; DppGal4
b
RFP
DAPI
hep r75;
UAS ey/UAS RFP; DppGal4
c
N=9
Fold change in intensity of RFP expression compared to UASey/UASRFP; DppGal4
hep r75; UASey/UASRFP; DppGal4
d
N=9
Fold change in area of RFP expression compared to UASey/UASRFP; DppGal4
hep r75; UASey/UASRFP; DppGal4
e
Dac
DAPI
UAS Bsk DN; UASey; DppGal4
f
Dac
DAPI
UAS Bsk DN; SerGal4/UAS ey
g
Dac
Eya
N=15
Fold change in area of expression compared to UASey; DppGal4
hep r75; UASey: DppGal4
UAS Bsk DN; UASey; DppGal4
h
Dac
Eya
N=15
Fold change in area of expression compared to SerGal4/UASey
hep r75; SerGal4/ UAS ey
UAS Bsk DN; SerGal4/ UAS ey
i
SerGal4/UASey
hep r75; SerGal4/UASey
Folds change in the level of transcript
***
dac

Figure S4

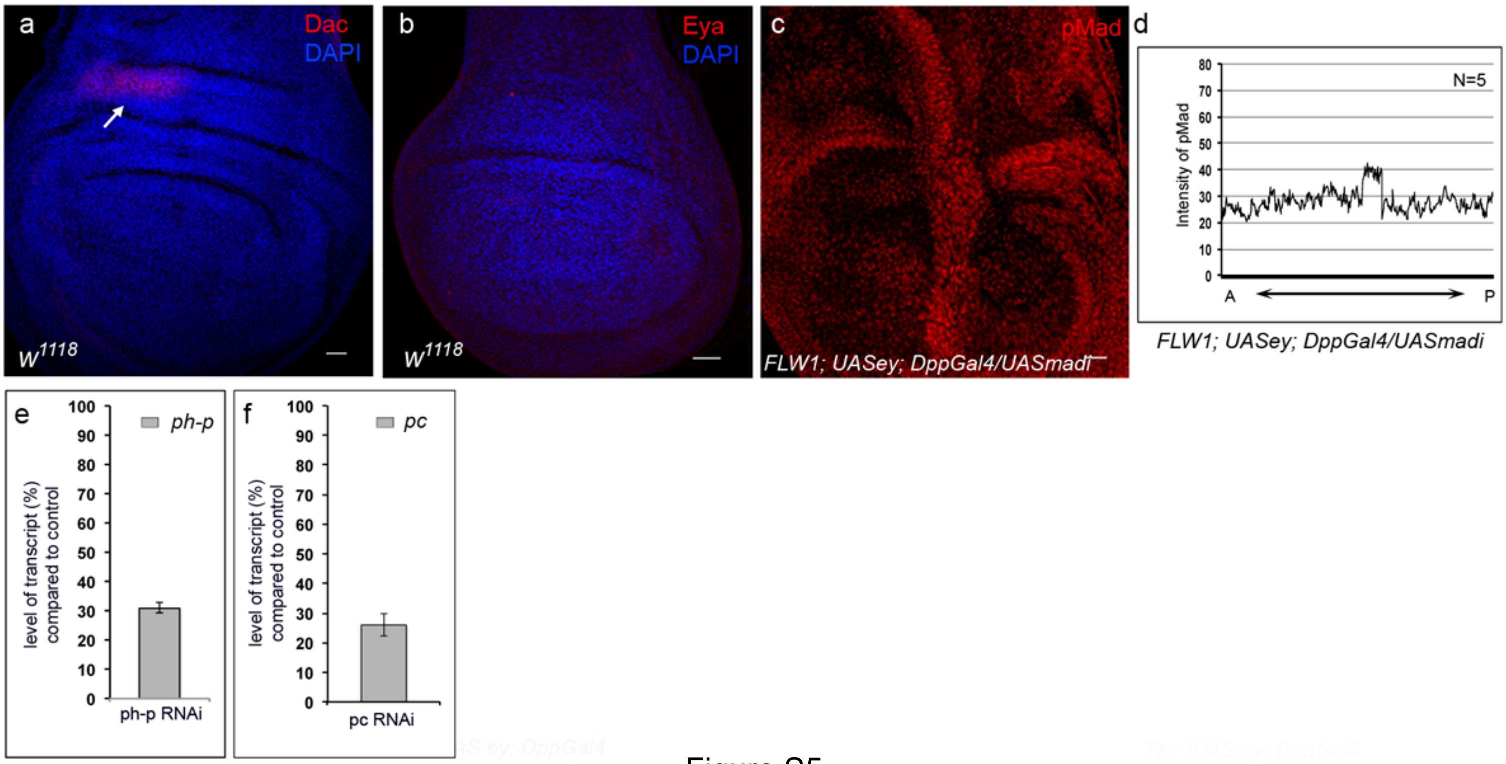
a
Dac
DAPI
W1118
b
Eya
DAPI
W1118
c
pMad
FLW1; UASey; DppGal4/UASmadi
d
N=5
Intensity of pMad
80
70
60
50
40
30
20
10
0
A
P
FLW1; UASey; DppGal4/UASmadi
e
ph-p
level of transcript (%) compared to control
100
90
80
70
60
50
40
30
20
10
0
ph-p RNAi
f
pc
level of transcript (%) compared to control
100
90
80
70
60
50
40
30
20
10
0
pc RNAi

Figure S5

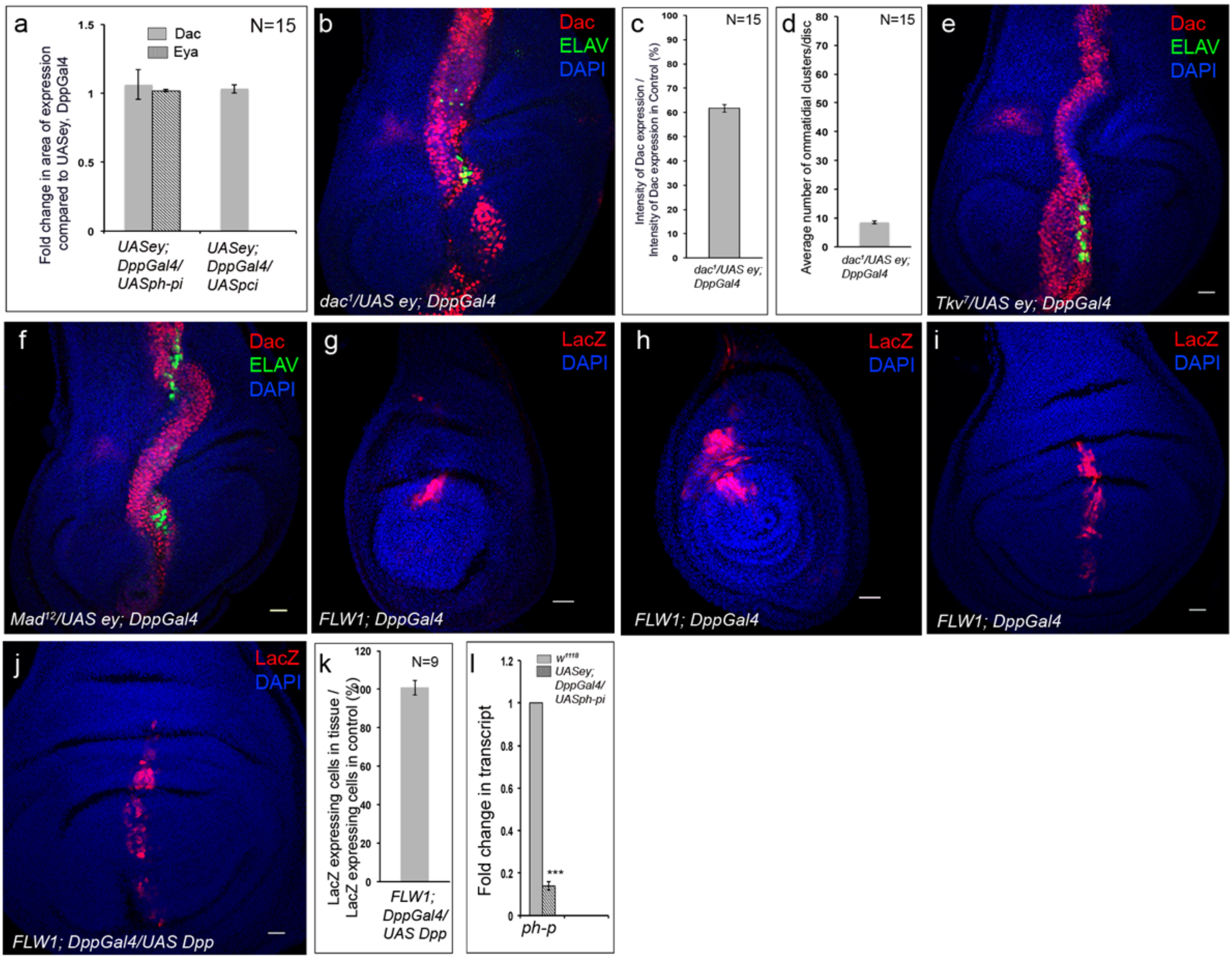
a
N=15
Dac
Eya
Fold change in area of expression compared to UASey, DppGal4
1.5
1
0.5
0
UASey; DppGal4/ UASph-pi
UASey; DppGal4/ UASpci
b
Dac
ELAV
DAPI
dac1/UAS ey; DppGal4
c
N=15
Intensity of Dac expression / Intensity of Dac expression in Control (%)
100
90
80
70
60
50
40
30
20
10
0
dac1/UAS ey; DppGal4
d
N=15
Average number of ommatidial clusters/disc
80
70
60
50
40
30
20
10
0
dac1/UAS ey; DppGal4
e
Dac
ELAV
DAPI
Tkv7/UAS ey; DppGal4
f
Dac
ELAV
DAPI
Mad12/UAS ey; DppGal4
g
LacZ
DAPI
FLW1; DppGal4
h
LacZ
DAPI
FLW1; DppGal4
i
LacZ
DAPI
FLW1; DppGal4
j
LacZ
DAPI
FLW1; DppGal4/UAS Dpp
k
N=9
LacZ expressing cells in tissue / LacZ expressing cells in control (%)
120
100
80
60
40
20
0
FLW1; DppGal4/ UAS Dpp
l
w1118
UASey; DppGal4/ UASph-pi
Fold change in transcript
1.2
1
0.8
0.6
0.4
0.2
0
***
ph-p

Figure S6

Supplementary Figure Legends:

Figure S1:

Genotypes are as mentioned. For all wing discs anterior is to the left. Dotted line marks the Dorsal/Ventral boundary of the wing disc.

(a) Changes in the level of *so* transcripts

(b) Level of *dTak1* transcript upon driving *dTak1RNAi*

(c) Level of *tkv* transcript upon driving *tkvRNAi*

(d-g) Ectopic expression of Dac and Elav in wing discs of genotypes mentioned.

(h,i) Ectopic expression of Eya in wing discs of genotypes mentioned.

(j) Quantification of the number of ectopic photoreceptor clusters.

(k) Quantification of the changes in areas of ectopic Dac and Eya expression.

(l) Quantification of the change in area of ectopic Dac expression.

(m) Change in the level of Dac transcripts.

Scale= 20μ

Figure S2:

Genotypes are as mentioned. For all eye discs posterior is to the bottom.

(a-e) Expression of Dac (a-c) and Eya (d-e) in eye discs of genotypes mentioned.

(f-h) Expression of Elav in eye discs of genotypes mentioned.

Scale= 20μ

Figure S3:

Genotypes are as mentioned. For all wing discs anterior is to the left.

(a) Level of *kayak* transcript upon driving *kayakRNAi*

(b-i) Ectopic expression of Elav in wing discs of genotypes mentioned.

(j) Quantification of the number of ectopic photoreceptor clusters.

(k-m) Ectopic puc-lacZ expression in wing discs of genotypes mentioned.

Scale= 20μ

Figure S4:

Genotypes are as mentioned. For all wing discs anterior is to the left. Dotted line marks the Dorsal/Ventral boundary of the wing disc.

(a,b) Reporter RFP expression for Dpp.

(c) Quantification of the changes in fluorescence intensity reporter RFP expression for Dpp.

(d) Quantification of the changes in area of reporter RFP expression for Dpp.

(e,f) Ectopic expression of Dac in wing discs of genotypes mentioned.

(g,h) Quantification of the changes in areas of ectopic Dac and Eya expression.

(i) Change in the level of Dac transcripts.

Scale= 20μ

Figure S5:

Genotypes are as mentioned. For all wing discs anterior is to the left.

(a) Expression of Dac in wild type wing discs.

(b) Expression of Eya in wild type wing discs.

(c-d) pMad expression in wing disc of genotype mentioned (c) and the corresponding average intensity profile (d).

(e) Level of *ph-p* transcript upon driving *ph-pRNAi*

(f) Level of *pc* transcript upon driving *pcRNAi*

Scale= 20μ

Figure S6:

Genotypes are as mentioned. For all discs anterior is to the left.

(a) Quantification of the changes in areas of ectopic Dac and Eya expression.

(b) Expression of ectopic Dac and Elav.

(c) Quantification of the change in fluorescence intensity of ectopic Dac expression.

(d) Quantification of the number of ectopic photoreceptor clusters.

(e,f) Ectopic expression of Dac and Elav in wing discs of genotypes mentioned.

(g,h) Expression of polycomb reporter lacZ in haltere (g) and leg (h) disc of genotypes mentioned.

(i,j) Expression of polycomb reporter lacZ in wing discs of genotypes mentioned

(k) Quantification of changes in area of polycomb reporter lacZ expression.

(l) Change in the level of ph-p transcript

Scale= 20μ

Table S1: Genotypes of flies used to screen for downstream target of dTak1 during ectopic eye formation

**JNK pathway**	
1	*UASbsk*^{DN}*; UASey; DppGal4*
2	*UASbsk*^{DN}*; UASey/SerGal4*
3	*hep*^{r75}*; UASey; DppGal4*
4	*hep*^{r75}*; UASey/SerGal4*
**P38 pathway**	
1	*UASey; UASp38aRNAi/DppGal4*
2	*UASey/SerGal4; UASp38aRNAi*
3	*UASey; UASp38bRNAi/DppGal4*
4	*UASey/SerGal4; UASp38bRNAi*
5	*UASp38b*^{DN}*; UASey; DppGal4*
6	*UASp38b*^{DN}*; UASey/SerGal4*
**IMD pathway**	
1	*UASey/UASIKKbRNAi; DppGal4*
2	*UASey; UASIKKbRNAi/DppGal4*
3	*UASey/SerGal4; UASIKKbRNAi*
4	*UASey/DmIKKg*^{Key1} *(Kenny1); DppGal4*
5	*UASey; rel*^{E20}*/DppGal4*
6	*UASey/SerGal4; rel*^{E20}

Table S2: Fly stocks used and genotypes of the lines used

Sl	Line	Genotype	Source
**Gal4 Driver lines**			
1	*Dpp-Gal4*	*w^{1118}; P{Gal4dpp.blk1}40C.6/TM6B,Tb^{1}*	Modified from BDSC Stock # 1553
2	*Ser-Gal4*	*w^{1118}; Ser Gal4/Ser Gal4*	BDSC Stock # 6791
**UAS responder lines**			
1	*UAS-ey*	*y^{1} w^{1118}; P{UAS-ey.H}UE11*	BDSC Stock # 6294
2	*UAS-bsk^{DN}*	*w^{1118}, UAS-bsk.DN*	BDSC Stock # 6409
3	*UAS-fos^{DN}*	*w^{1118}; UAS-$Dfos^{bZIP}$*	BDSC Stock # 7215
4	*UAS-Dpp*	*w^{1118}; UAS-Dpp*	BDSC Stock # 1486
5	*UAS-RFP*	*w^{1118}; P{UAS-RFP.w}2*	BDSC Stock # 30556
**Reporter lines**			
1	*Dpp-lacZ*	*w^{1118}; Dpp-lacZ/CyO*	Blackman et al., (1991)
2	*Puc-lacZ*	*w^{1118}; puc-LacZ/TM3,Ser*	Ring & Martinez-Arias (1993)
3	*FLW1*		Cavalli & Paro (1998)
**Mutant lines**			
1	*hep*	*w^{1118}, hep^{r75}/FM7c*	BDSC Stock # 6761
2	*mad*	*w^{1118}; Mad^{12}P{neoFRT}40A/CyOAct-GFP*	Modified from BDSC Stock # 58785
3	*tkv*	*tkv^{7}, cn^{1}, bw^{1}, sp^{1}/CyOAct-GFP*	Modified from BDSC Stock # 3242.
4	*dac*	*b^{1}, dac^{1}, pr^{1}, cn^{1}, wx^{wxt}, bw^{1}/CyOAct-GFP*	Modified from BDSC Stock # 4273
5	*psc*	*cn^{1}, Psc^{1}, bw^{1}, sp^{1}/CyOAct-GFP*	Modified from BDSC Stock # 4200
6	*psc*	*PSc^{h27}/CyO*	BDSC Stock # 5547
7	*Tak1*	*y^{1} w^{1118}, $Tak1^{2}$*	BDSC Stock # 26272
8	*Tak1*	*w^{1118}, $Tak1^{179}$*	BDSC Stock # 26275
9	*foxo*	*w^{1118}; $foxo^{del94}$/TM6B,Tb*	BDSC Stock # 42220

Mutant Lines	Genotypes of lines used for this study
y^{1} w^{1118}, Tak1(2)	*$TAK1^{2}$/ y; UASey/+; DppGal4/+* (male)<br>*$TAK1^{2}$/ y; UASey/+; DppGal4/UASDpp* (male)<br>*$TAK1^{2}$/ y; UASey/+; DppGal4/PuclacZ* (male)<br>*$TAK1^{2}$/ y; UASey/ Ser Gal4; +/+* (male)<br>*$TAK1^{2}$/ y; UASey/Ser Gal; PuclacZ* (male)<br>*$TAK1^{2}$/ y; UASey/Ser Gal; UASDpp/+* (male)
w^{1118}, hep^{r75}/FM7c	*hep^{r75}/+; UASey/+; DppGal4/+*<br>*hep^{r75}/+; UASey/+; DppGal4/UASDpp*<br>*hep^{r75}/+; UASey/ Ser Gal4; +/+*<br>*hep^{r75}/+; UASey/Ser Gal; UASDpp/+*<br>Since male larvae exhibited lethality, we used heterozygous female larvae for our analyses.

w^{1118};Mad^{12}P{neoFRT}40A/CyOAct GFP	*w^{1118}; Mad^{12} P{neoFRT}40A/Cyo/UASey; DppGal4/+*<br>*w^{1118}; Mad^{12} P{neoFRT}40A/Cyo/UASey; DppGal4/PuclacZ*
Tkv^{7}/CyOAct-GFP	*w^{1118}; Tkv^{7}/UASey; DppGal4/+*<br>*w^{1118}; Tkv^{7}/UASey; DppGal4/ PuclacZ*
Dac^{1}/CyOAct-GFP	*w^{1118}; Dac^{1}/UASey; DppGal4/+*
Psc^{1}/CyOAct-GFP	*w^{1118}; Psc^{1}/UASey; DppGal4/+*
PSc^{h27}/CyO	*w^{1118}; PSc^{h27}/UASey; DppGal4/+*<br>*TAK1(2)/ y; PSc^{h27}/UASey; DppGal4/+* (male)
w^{1118};FoXo^{del94}/ TM6B,Tb	*w^{1118}; UASey/ FoXo^{del94}; DppGal4/+*<br>*w^{1118}; UASey/ FoXo^{del94}; SerGal4/+*
w^{1118}, UAS-bsk.DN	*w^{1118}, UAS-bsk.DN / y; UASey/+; DppGal4/+* (male)<br>*w^{1118}, UAS-bsk.DN / y; UASey/+; Ser Gal4/+* (male)

Table S3: List of primary and secondary antibodies used.

Antibody	Dilution	Source
**Primary**		
mouse α-Dacshund	1:30	Developmental Studies Hybridoma Bank, Iowa
mouse α-Eyes absent	1:2	Developmental Studies Hybridoma Bank, Iowa
rat α-ELAV	1:100	Developmental Studies Hybridoma Bank, Iowa
rabbit α- β- galactosidase	1:1000	Molecular Probe, USA
mouse α-β- galactosidase	1:100	Promega, USA
rabbit α- p-Mad	1:2000	Dr. E. Laufer, Columbia University
**Secondary**		
Cy^{TM3}-Conjugated donkey anti rabbit	1:600	Jacksons Immuno Research Laboratories, USA
Cy^{TM3}-Conjugated goat anti mouse	1:600	Jacksons Immuno Research Laboratories, USA
(FITC)-Conjugated goat anti mouse	1:600	Jacksons Immuno Research Laboratories, USA
(FITC)-Conjugated donkey anti rat	1:600	Jacksons Immuno Research Laboratories, USA

Table S4: List of primers used

Gene	Primer Sequence	
dTak1	Forward	5’ CTCGGGCCAACTGGACAATA 3’
	Reverse	3’ ATCAAAGCCATTCGCCTCCA 5’
dacshund (*dac*)	Forward	5’ CTCCAGGAATTGCTCTCCCA 3’
	Reverse	3’ TCGTCCTCGACGCCGATTAT 5’
eyes absent (*eya*)	Forward	5’ ATCGTGGAAATGTTGGCACC 3’
	Reverse	3’ GGAGGTTACCAGCACGTTGA 5’
sine oculis (*so*)	Forward	5’ GTGTTTGCGAGGTTCTCCAGC 3’
	Reverse	3’ AATGCGCTTTCAACCACAGG 5’
polyhomeotic proximal (*php*)	Forward	5’ CAGACCCAGCAAAACCAGATT 3’
	Reverse	3’ TCCATTGTGGGGCATGACAG 5’
poly comb (*pc*)	Forward	5’ GAGTAAGGGGAAGTTGGGGC 3’
	Reverse	3’ TCCACGACGCCCTTCTTAAC 5’
thickveins (tkv)	Forward	5’TTCTCATGTGCAAGGTAGCCG3’
	Reverse	3’GCGTCGCTTGTAGGTGAAAC5’
kayak (kay)	Forward	5’GACCGATACTTCAAGTGCCCA3’
	Reverse	3’TTGAGGTATTCGCGTTGCTG5’
